# Adolescent Involvement in Co‐Authoring Peer Reviewed Publications: A Reflection of Challenges and Best Practice Recommendations

**DOI:** 10.1111/hex.70597

**Published:** 2026-03-04

**Authors:** Rebecca Raeside, Allyson R. Todd, Sisi Jia, Sara Wardak, Khalid Muse, Kevin Kapeke, Surabhi Dogra, Emma Soo, K. Connor, Molly O'Sullivan, Christina Zorbas, Stephanie R. Partridge

**Affiliations:** ^1^ Faculty of Medicine and Health, Susan Wakil School of Nursing and Midwifery The University of Sydney Sydney Australia; ^2^ Charles Perkins Centre The University of Sydney Sydney Australia; ^3^ Global Centre for Preventive Health and Nutrition, Institute for Health Transformation Deakin University Geelong Australia; ^4^ The Victorian Health Promotion Foundation (VicHealth) Melbourne Australia; ^5^ Emerging Professionals Network International Association for Adolescent Health Sydney Australia; ^6^ Faculty of Medicine and Health, The Health Hive, Susan Wakil School of Nursing and Midwifery The University of Sydney Sydney Australia; ^7^ Global Adolescent Health Murdoch Children's Research Institute Melbourne Australia

## Abstract

Adolescents (10–24 years old) are increasingly being included as research partners or co‐researchers in line with the United Nations Convention on the Rights of the Child. However, there is no existing guidance that explores the nuance of how adolescents can be involved as co‐authors in peer reviewed research publications. Through this article, we reflect on our experiences co‐authoring peer reviewed publications with adolescents and support this by drawing upon empirical evidence. This Viewpoint article discusses challenges before, during and after publication and provides guidance to journals, publishers, and researchers on ethically and authentically involving adolescents as co‐authors. We also propose best practice recommendations to support co‐authorship with adolescents. This article is intended to create a more inclusive publication landscape for adolescents who are increasingly participating as co‐researchers across health research and should rightly be included as co‐authors. Patient and Public Contribution: This article was written in collaboration with adolescents, professionals and academics contributing equally. All authors contributed to article planning, investigation, reviewing and editing and approving the final version of the article.

## 1 Introduction

Globally, there is significant traction on consumer involvement and participatory methods in health research, especially among research with adolescents*. Adolescent involvement is defined as ‘research being carried out “with” or “by” adolescents rather than “to”, “about” or “for” them’. [1] Since adoption of United Nations Convention on the Rights of the Child (UNCRC, 1989) [2], there has been a growth in research with adolescents that promotes and protects human rights by involving them as active research team partners (e.g. co‐researchers). This rights‐based approach to health research increases research quality and accessibility, building capacity for agency and leadership amongst this generation [3]. Throughout the literature, there is a breadth of reporting on the positive impacts and challenges to adolescent research involvement. Positive impacts include to the research, the adolescents involved, and the researchers (e.g. enhanced study design, increased knowledge and skill development) [4]. However, multiple challenges exist including organisational constraints, researchers’ preparedness, methodology for adolescent involvement, ethical involvement and adolescent specific issues [5]. In addition, reporting and evaluation of adolescent contributions are often ambiguous [6], leading to challenges in advancing this field of research.

*In this article, we define adolescents as those aged 10–24 years, aligning with the 2016 and 2025 *Lancet* Commissions on Adolescent Health and Wellbeing [7, 8, 9]. We acknowledge the use of other similar terms interchangeably, such as ‘youth’ and ‘young people’ across evidence sources.

Recent publications aim to bridge this research gap through checklists and guidelines to enhance adolescent involvement in research. The 2025 *Lancet* Commission on Adolescent Health and Wellbeing incorporated a checklist for reporting research with adolescent and youth engagement [10]. Additionally, Seidl and colleagues proposed a framework for co‐research with children and adolescents across six themes, including the need to give appropriate recognition for their contributions [11]. While these tools are useful for reporting adolescent involvement and co‐research more broadly, there remains a lack of clear guidance on how to involve adolescents in co‐authoring peer reviewed publications (herein referred to as publications). Authorship on publications is granted to individuals who make significant intellectual or scholarly contributions to the work. This standard has important implications for maintaining integrity and accountability of the research presented. The International Committee of Medical Journal Editors (ICMJE) recommends that authorship is based on four criteria (Table 1) with those fulfilling the first criterion encouraged to have the opportunity to satisfy the rest [12].

**Table 1:** ICMJE authorship criteria.

1. Substantial contributions to the conception or design of the work or the acquisition, analysis, or interpretation of data for the work; 2. Drafting the work or reviewing it critically for important intellectual content; 3. Final approval of the version to be published; 4. Agreement to be accountable for all aspects of the work in ensuring that questions related to the accuracy or integrity of any part of the work are appropriately investigated and resolved.

Adolescents (10–24 years old)* are becoming increasingly engaged as co‐researchers or co‐contributors to research (e.g., co‐design, youth advisory groups, citizen science) related to criterion one of the ICMJE, which is common in line with the UNCRC [3, 13]. Therefore, they have a right to be provided with the opportunity to be co‐authors and meet criteria 2–4. There is little guidance on how these existing criteria can apply to the meaningful involvement of adolescents as co‐authors in peer‐reviewed research publications.

A previous review synthesised studies which involved patients in preparing and disseminating health research publications [14], yet no included studies captured adolescent populations. Guidance for patients and researchers on whether patients should be a co‐author or acknowledged in publications has also been published [15]. However, these previous studies do not capture or discuss the nuanced challenges to involving adolescent populations. This guidance is imperative to ensure the integrity of research conducted in partnership with adolescents is maintained, and foster opportunities which build their capacity as future leaders. Below, we reflect upon our experiences of co‐authoring research publications with adolescents and draw upon existing evidence. We reflect on challenges encountered before, during and after publication, and provide guidance on ethically and authentically involving adolescents as co‐authors on peer‐reviewed publications. We also propose best practice recommendations to support co‐authorship with adolescents.

## 2 Before Publication

### 2.1 Adolescent Participation and Power Dynamics

Adolescent research involvement as co‐authors and thus co‐researchers cannot be discussed without considering power imbalances [5, 16]. Adolescent involvement can take many forms, from tokenistic to youth‐initiated, where adolescents lead and share decision‐making with adults [17]. To avoid tokenism, adolescent involvement requires changes in power structures that enable adolescents to be considered ‘experts’ in their own needs and realities, while building their leadership capacities [18]. Adolescents can participate in research (beyond being a participant) through different modes according to the Lansdown‐UNICEF Conceptual Framework [19] from consultative, collaborative and adolescent‐led. Under these participation modes, adolescents may contribute to stages of the research cycle, in line with criteria one of the ICMJE Guidelines. A scoping review which investigated adolescent participation in transforming healthy food environments included a youth advisory group as co‐authors, as they were consulted at each stage of the scoping review [20]. One adolescent co‐author from this Viewpoint described the process as ‘eye‐opening, but also intimidating at first’ capturing the vulnerability and empowerment that comes with working in public health research. Furthermore, adolescents engaged as co‐researchers in chronic disease prevention research highlighted being able to communicate data and perspectives through co‐authoring research publications is key to avoiding tokenistic participation [21]. Research also demonstrates authentic and meaningful participation as co‐researchers enhances adolescents’ leadership and life skills [22], may lead to better policies, and provides greater trust between adolescents and authorities [19]. This in turn facilitates adolescents to become agents of change in their communities. This extends to co‐authoring publications, which holds potential to enhance adolescents’ future prospects by providing them with reputable platforms to have their contributions formally recognised, demonstrate thought leadership, contribute to public and political debates and reduce tokenistic research participation.

### 2.2 Planning for Inclusion

For co‐authorship with adolescents, academics must plan for their inclusion. There are multiple existing guidelines which assist in preparation and planning for adolescent involvement in health research [23], which rarely extend to discuss co‐authorship. Drawing from our experiences, we advise that academics consider the resources they have available to support adolescent participation in co‐authoring. This includes being able to hold regular meetings (in‐person or online) which provide education on ICMJE guidelines, publishing processes, and the responsibilities of being an author. In addition, it is recommended there is a designated person for adolescents to contact for support and queries during the publication process, including drafting and reviewing of publications (e.g., coordinator, mentor). This assists with building rapport and allowing open and honest communication between adolescents and adult researchers. Yet adolescents should also be provided flexibility in the methods to provide feedback (e.g., email, online or in‐person meetings). Finally, researchers must also be able to remunerate adolescents appropriately for their contributions. We have experienced issues with institutional payroll processes for compensating adolescents effectively and efficiently; for one‐off or repeated engagements, and when engaged in an ongoing role. Fair compensation for adolescents’ time and expertise is necessary and should be adjusted for an individual's context, proportionate to their economic and social situation and overall level of involvement in the research [5, 24].

### 2.3 Clear Authorship Practices

To advance adolescent co‐authorship, they must only be listed with a complete understanding of authorship and the responsibilities involved [25]. Within research teams, publication plans are often circulated to members for transparency on involvement in publications, the author order, and who is acknowledged. The same should be provided to adolescent co‐authors. This could be in the form of a Terms of Reference agreement which outlines their roles and expectations within the publication process, including timelines they will be required to engage with the research team for writing and reviewing manuscripts. There are also the complexities of token and gift authorship. Tokenistic inclusion of adolescents within research is an ongoing concern [26, 27]. Gift authorship and associated terms indicates when a co‐author has completed little to no work on the manuscript yet is still a named co‐author [25]. Gift authorship among adolescents has been noted within the literature [28], especially to improve chances of tertiary education. Yet, engaging adolescent co‐authors through gift or tokenism goes against ICMJE guidelines, where authors must agree ‘to be accountable for all aspects of the work’.

While it is best practice to involve adolescents from the research conception, it is recognised this is not always possible due to available resources. Academics have a responsibility to ensure that if they are involving adolescents, clear processes must be established for recognition of their contributions. Adolescents may contribute to research but not meet ICMJE criteria—and in this case, they should be appropriately acknowledged instead. Nagata et al. have developed a checklist for reporting research with adolescent and youth engagement [10]. When adolescents are engaged throughout the research process, this checklist provides a framework for appropriate reporting throughout the manuscript. When adolescents are only involved in some stages, the checklist may serve as a tool to assess the level and nature of involvement, and whether co‐authorship or acknowledgement is appropriate.

## 3 During Publication

### 3.1 Communication With Co‐Authors and Journals

During the process of publication, adolescents must be kept up to date with access to documents and information on how they will be credited within the publication, submission to the journal, receiving reviewer comments, acceptance and publication. First, it is vital to ensure adolescents are comfortable with the way they are acknowledged within the publication. In our experience, journals often request identifiable information about adolescent co‐authors to ensure they are true co‐researchers. This must be carefully balanced against adolescents’ privacy, especially if they are from a marginalised group or with lived experience of a health condition [15, 29]. Our experience has led to crediting of youth in different methods, including as named co‐authors [30], using a group author name [20], or their preferred name, and including positionality statements [31]. Second, adolescents who have grown up in a digital world, are used to large amounts of information being disseminated at a rapid pace. It is important to communicate to them that scientific publishing is a slower process, going through a thorough peer review process from experts in the field. Regular updates on the status of the manuscript are important, especially to keep them engaged and provide feedback once reviewer comments are received.

The current publication landscape is not inclusive of adolescents as co‐authors in research, even when they meet ICMJE authorship criteria. We have had to advocate for adolescent co‐authors and address concerns of journals and peer reviewers, many of whom believe publications belong to the domain of traditional ‘adult experts’ and are more important to adult academics than adolescents. In our experiences, adolescents understand and appreciate the value that exists for them personally and professionally when they are warranted the opportunity to write for academic journals [21], and this is also reflected within the literature [15, 32]. To create more equitable and inclusive landscapes, there is a need to navigate power imbalances that systematically exclude adolescents’ written contributions to research, and advocate for their inclusion in academic publishing.

### 3.2 Equity Considerations

Our experiences to date have highlighted several administrative and regulatory hurdles from academic journals that hinder co‐authoring with adolescents. Article processing fees for publishing in academic journals can make this method of dissemination financially inaccessible to adolescents without institutional support. Journals could look to adopt a method of fee waiver for adolescent‐led publications, similar to of low‐ and middle‐income countries. Additionally, journal requirements for details like institutional email addresses, affiliations, using first names and surnames, listing academic degrees, and limits on author numbers hinder appropriately crediting adolescent co‐authors. Online journal systems are often not flexible, with all details required during submission. Lack of flexibility in providing these details can cause delays to publication or restrict submissions to certain journals. In some cases, publishers do not allow non‐academics to write for them, excluding adolescents from the process entirely.

Our reflections on journal requirements have been detailed previously [33]. However, these adolescent co‐researchers were 11–13 years old, which may have additional ethical complexity in receiving appropriate credit for their contributions. We acknowledge the distinction between children and adolescents is important for ethical considerations relating to co‐authoring publications. Additional challenges arise with co‐authors who are under 18 years old. Journal editors and peer reviewers often request ethical approval and parent or guardian consent for involving adolescent co‐authors assuming they are the ‘subject of the research’ rather than co‐researchers [5, 34]. Depending on context, ethical approval may not be required for adolescents to be engaged as co‐authors. However, consent to involve adolescents in the co‐authoring process should always be sought, yet not in the formal sense that journal editors or peer reviewers are often requesting. A paradigm shift is needed where adolescent co‐researchers are viewed as powerful contributors to research and can play an important role as conduits for other adolescent voices [34]. Bioethical principles support this conclusion, where an adolescent possesses capacity to make decisions about their participation, that should be respected and upheld [35]. Regardless of age, guidelines to protect youth wellbeing through participation have been developed and are now available [36] and relevant ethical guidelines must be followed.

## 4 After Publication

Following publication, it is vital to involve adolescent co‐authors in the dissemination of knowledge, especially in youth friendly formats. This may include social media content, videos, advocacy through youth networks and events, including adolescents within media opportunities, conference or webinar presentations, and inviting them to develop plain language summaries and write blogs. It is also important to address practical challenges following publication with adolescent co‐authors. This includes supporting them to create an ORCID account if interested, and education on predatory journals who may send emails post publication. It is important to keep adolescents engaged beyond publication. This can be achieved in multiple ways including providing updates on impacts of the study (e.g. publication cites in national or international reports or policy) and plans for follow‐up studies. However, adolescent wellbeing should always be upheld to ensure ongoing opportunities do not lead to burn out. Finally, adult researchers should seek to evaluate their process of including adolescent co‐authors to understand what worked well and what can be improved for future involvement.

Despite the challenges noted, there are positive examples of adolescent leadership in academic publishing, including *The Lancet Child and Adolescent Health* Reflections (welcomes reflections from adolescents), Young Voices articles in *BMJ Pediatrics Open* (commissioned short articles from individual young people or organisations they represent), the *Future Healthy Countdown* (encourages authorship for those aged under 30 years), and the Second *Lancet Commission* on Adolescent Health and Wellbeing, where youth commissioners played an integral role.

## 5 Recommendations and Conclusion

Based on our experiences and available evidence, we provide recommendations in Table 2 to journals, publishers and researchers to ethically and authentically involve adolescents in co‐authoring publications and create a more inclusive landscape.

**Table 2:** Recommendations for co‐authoring peer‐reviewed research publications with adolescents.

	Recommendation	Practical examples
Before publication	All actors—provide more inclusive participation opportunities for adolescents to co‐author publications [5, 37] Researchers—include adolescents who can provide diverse and meaningful contributions to research projects and publications in line with their capacity and/or lived experience [36] Researchers—seek adolescent consent in an informal manner to involve them within the co‐authoring process and set expectations	Ensure adolescent research roles are transparent e.g. consultive, collaborative, adolescent‐led Hold regular meetings to provide education on ICMJE guidelines, publishing processes, authorship responsibilities Provide flexible methods for adolescents to give feedback on manuscripts Have a designated contact person to act as a conduit between the researchers and adolescents
During publication	Researchers—ensure participation focuses on building leadership and research capacity through mentorship and education, in line with available resources [4, 22, 38] All actors—advocate for shifts in power to reduce system‐level barriers [16, 38] Journals—requirement of evidence (e.g. statement, checklist) that meaningful adolescent involvement in the research process occurred [9]	Efficient and adequate compensation for adolescent co‐researchers, in line with contributions (consult national organisations for guidelines on compensation as this may be country specific) Regular updates to adolescent co‐authors throughout the peer review process Advocate for shifts in journal and publishing requirements to be less prohibitive and allow authors from outside of academic institutions, including appropriate crediting of adolescent co‐authors
After publication	Researchers—include adolescent co‐researchers in the dissemination process Researchers—maintain engagement with adolescent co‐authors beyond publication (e.g. via mentoring) All actors—involve adolescents in the process of translating research into policy and practice (where appropriate) Journals—review or develop processes for adolescent co‐authors, ensure inclusive and streamlined processes	Provide equitable opportunities for co‐creation of dissemination materials (e.g. social media content, videos, summaries) Invite and support adolescents to co‐present findings at conferences or webinars, media opportunities (ensure to seek consent) Provide ongoing communication with adolescent co‐authors on impact of research and follow‐up studies Provide opportunities for ongoing engagement with research, carefully balance this with overburden which can lead to burn out

Adolescents have a right to co‐author research publications that align with their contributions, lived experiences and expertise. From our experiences and the literature, we provide guidance to researchers and publishers on the nuances of meaningfully involving adolescents as co‐authors before, during, and after publication, filling a critical gap in the literature. We conclude that adolescents should be provided support and education throughout the co‐authoring process to build their capacity, researchers must advocate for their inclusion in adult‐centric spaces, particularly in discourse involving young people's interests, and journals must develop processes that provide equitable opportunities. Despite some progress, there is still considerable advancement needed to provide an inclusive publishing landscape for adolescents on research that impacts them.

## Author Contributions

This work originated from the authors' personal experiences of including adolescents as co‐authors on research publications, and barriers faced when trying to do so. Sara Wardak, Khalid Muse, Emma Soo and K. Connor are all adolescents who have first‐hand experience in academic publishing. Rebecca Raeside, Allyson R. Todd, Sisi Jia, Kevin Kapeke, Surabhi Dogra, Molly O'Sullivan, Christina Zorbas and Stephanie R. Partridge are academics and professionals who have expertise in adolescent consumer engagement in research. This article was written in collaboration with adolescents and academics contributing equally. Rebecca Raeside led conceptualisation and writing of the original draft. All authors contributed to article planning, reviewing article drafts, providing critical feedback and approving the final version of the article.

## Ethics Statement

The authors have nothing to report.

## Consent

The authors have nothing to report.

## Conflicts of Interest

The authors declare no conflicts of interest.

## Positionality Statement

The authorship team comprises 9 females, 2 males and 1 gender diverse person. The authors hold a range of professional roles including researchers (PhD students, EMCRs and senior researchers), public health consultants, public health professionals, policy and government relations, and young people. Their focus areas include adolescent health, public health, nutrition, law, health equity, environmental health, digital health. Their diverse experiences and perspectives relating to co‐authoring peer reviewed research publications are captured and represented in this Viewpoint article.

## Supporting information


**Appendix 1:** Completed checklist for reporting research with adolescent and youth engagement.

## Data Availability

The authors have nothing to report.
