# Supplementary material for: Adolescent Involvement in Co‐Authoring Peer Reviewed Publications: A Reflection of Challenges and Best Practice Recommendations
Source: Health Expect. 2026 Mar 4;29(2):e70597. doi: 10.1111/hex.70597 (PMC12961158; doi:10.1111/hex.70597)
Supplement: Supplementary file 1 — Appendix 1: Completed checklist for reporting research with adolescent and youth engagement. [file HEX-29-e70597-s001.docx]

**Appendix 1:** Completed checklist for reporting research with adolescent and youth engagement

| **Authorship** | All authors were involved in the planning of the article, critically reviewing drafts of the manuscript and providing approval of the article for publication. | |
| --- | --- | --- |
| **Acknowledgements** | Thank you to the 32 adolescents who have taken part in the Health Advisory Panel for Youth at the University of Sydney since 2021. They have participated in discussions and had firsthand experiences of challenges around adolescent co-authorship of peer-reviewed publications. | |
| **Aim** | Through our previous research and experiences, we have experienced significant challenges with involvement of adolescents in co-authoring peer reviewed publications with adolescents. Previous research does not capture or discuss the nuances of involving adolescents in co-authoring peer-reviewed publications. Here, we build off previous work by reflecting on our own challenges, including the lived experience of adolescents who have been involved, and draw upon existing evidence to provide recommendations. | |
| **Methods** | Adolescents engaged | 4 adolescents who are younger than 24 years are listed as co-authors, as well as 2 early career professionals who have experience contributing to past youth health research, including co-authoring peer-reviewed publications. |
|  | Stages of involvement | Adolescents were involved throughout the entire process, equal to adult researchers. Initially, adolescents provided input into the article outline, including input on the recommendations arising. All adolescent co-authors participated in several rounds of review and refinement of the article and recommendations. |
|  | Level or nature of involvement | Adolescents were engaged in a collaborative mode of participation throughout the process. Their roles included providing critical input into the article text, development and refinement of recommendations and reviewing and editing of the final article for publication. |
| **Discussions and conclusions** | Overall influence | This article would not be possible without the contributions of adolescent co-researchers. While the article was conceptualised by adult researchers, adolescents had input into all aspects of the article. |
|  | Critical reflection | While article conceptualisation was by adult researchers, the article has been inclusive in nature from the beginning – where article outlines have been shared with all co-authors for comment. Future efforts could aim to include broader networks of adolescents to ensure that their experiences are captured. However, due to time and resource constraints we were limited to adolescent co-researchers within our networks. |
